# Slipping Through the Gap: Exploring the Influence of Social Health Insurance on Access to Healthcare for Older Migrant Workers

**DOI:** 10.3389/ijph.2024.1606655

**Published:** 2024-03-13

**Authors:** Chengxu Long, Wei Yang, Shangfeng Tang

**Affiliations:** ^1^ Department of Global Health and Social Medicine, King’s College London, London, United Kingdom; ^2^ School of Medicine and Health Management, Huazhong University of Science and Technology, Wuhan, Hubei, China

**Keywords:** older migrant workers, social health insurance, access to healthcare, migrant administration system, migrant health

## Abstract

**Objectives:** Older migrant workers (OMWs) frequently confront barriers to accessing care, as their Social Health Insurance (SHI) coverage may not extend beyond their hometown. This study seeks to investigate whether Chinese OMWs can still derive benefits from SHI in accessing healthcare services, even when their SHI is not registered in the same location as their current residence.

**Methods:** This study used data from 2015 China Migrants Dynamic Survey and focused on OMWs aged 60 years and older (*N* = 3,050). Logistic regression models were employed to investigate the factors influencing healthcare use.

**Results:** Having SHI registered in current place of residence and interprovincial migration were significantly associated with increased likelihoods of doctor visits among OMWs. However, inpatient services use did not appear to be associated with the SHI registration place and migration range.

**Conclusion:** Chinese OMWs derive fewer benefits from SHI in accessing healthcare services when their SHI is not registered in current residence. Governments in Low- and Middle-Income Countries should consider implementing targeted policies to provide adequate protection for OMWs and expand the coverage of direct reimbursement for cross-province healthcare services.

## Introduction

Globally, the number of migrant workers continues to rise. Despite a substantial portion of older migrant workers (OMWs) actively remaining part of the labor force and earning income to sustain their families even post-retirement age [1], the majority of these OMWs predominantly engage in unskilled and physically demanding occupations. These roles are often characterized by limited job security and adverse working conditions [2]. Consequently, OMWs may face a heightened risk of exposure to hazardous substances, occupational diseases, and workplace injuries [3]. Given that most OMWs are from disadvantaged socioeconomic backgrounds and may not have enrolled in social security programs within their current places of residence, they are confronted with legal and policy barriers that hinder their access to healthcare services and public welfare benefits, particularly when compared to native or local populations [4].

China hosts one of the world’s largest populations of migrant workers. In 2021, the country had approximately 292.5 million migrant workers, constituting over 20% of the total population [5]. These migrant workers are often at a higher risk of health issues due to engaging in strenuous manual labor, frequently working in poor conditions, or lacking access to healthcare services [6]. While a majority of the Chinese population is entitled to various welfare benefits, including Social Health Insurance (SHI), access to these benefits is contingent upon one’s *Hukou* status—a legal document tied to an individual’s household registration based on their birthplace [7]. This policy feature may pose negative effects for Older Migrant Workers (OMWs), as the majority of rural-to-urban OMWs are covered by the SHI registered in their hometown rather than their current place of residence. If the rights and entitlements of SHI benefits are strictly confined to OMWs’ places of birth, it poses a significant challenge as many may encounter barriers in accessing healthcare when needed.

Based on the discussion above, this study examines the effect of different SHI registration locations on access to healthcare among OMWs. Particular attention is given to the variable of migrant range, investigating how the distance an OMW is from their hometown influences access to healthcare. This analysis is crucial as, in certain provinces, SHI schemes extend coverage to services beyond the OMWs’ birthplace, granted they seek care within the province of their birth. Data are drawn from the China Migrant Dynamic Monitoring Survey 2015, and the analyses are based on logistic regression. In light of the global aging trend and the increasing population of OMWs within and across countries, our targeted measures provide valuable insights for policymakers grappling with similar challenges in the global community. This study would also provide insights on tailored healthcare policies aimed at supporting vulnerable OMWs in Low- and Middle-Income Countries (LMICs). In this article, we begin by introducing the barriers to accessing healthcare services among OWMs, followed by an overview of SHI and a literature review on its impact on OMWs. Next, we outline our data and methods, present our results, and conclude with a discussion of the reasons behind our findings, along with their implications and limitations.

### Barriers to Healthcare Services Access Among Older Migrant Workers

Migrant workers often engage in exploitative work with heavy workloads and limited wages [8]. They are more likely to be exposed to hazardous working conditions [9], which are known to contribute to poor health outcomes among migrant workers [4]. Hansen and Donohoe [10] state that migrant workers have insufficient labor protection measures and a higher risk of workplace injuries and abuse. OMWs have the dual characteristics of being both migrant workers and older adults. The majority of OMWs come from low- and middle-income areas [11]. They are more disadvantaged in terms of socioeconomic status and access to healthcare compared to younger migrant workers [12]. Simultaneously, OMWs face significant barriers to maintaining employment and experience multiple forms of discrimination based on factors like age and disability [12]. Existing studies have documented that migrant workers are underserved by the healthcare system in their current place of residence and lack sufficient social protection for their health and wellbeing [13–15]. However, these studies have not focused on OMWs. This study seeks to bridge the research gap to understand the impact of the registration place of SHI on access to healthcare for OMWs.

Internationally, migrant workers often face significant barriers to accessing health services and restrictions on their entitlement to statutory health services [16, 17]. Liem [18] found that international migrant workers often lack adequate health insurance in their host countries. Siddiqi [19] claimed that racialized migrants in Canada are more likely to lack a regular source of healthcare and receive fewer needed health services than native-born individuals. Research in Europe shows that immigrants face significant disadvantages in accessing needed health services due to social, linguistic, and cultural barriers [20]. Azzopardi-Muscat [21] states that Europeans have limited entitlements to cross-border healthcare benefits, as patients who receive health services outside their home country must reimburse healthcare costs according to the health insurance program in their home country.

### Social Health Insurance and Older Migrant Workers in China

China has an established Social Health Insurance (SHI) system and a growing number of OMWs. The number of Chinese migrants has increased from 154 million in 2010 to 376 million in 2020 [22]. A report by the China National Health Commission suggested that seeking better employment opportunities was one of the main reasons for migration among older migrants [23]. Additionally, the proportion of older adults over 65 years old in China has increased from 10% in 2015 to 13% in 2021 [24], indicating that the number of OMWs will continue to rise.

China’s SHI system includes schemes such as the New Rural Cooperative Medical Scheme (NCMS), Urban Residences Insurance (URI), and Urban Employee Residences Insurance (UEI), which separately cover rural residents, urban residents with formal employment, and urban residents without formal employment [25]. The Chinese government initiated the Urban and Rural Health Insurance (URHI) program by consolidating NCMS and URI in 2016, although progress has been uneven [25]. The type of SHI available to Chinese individuals is determined by the household registration system (*Hukou* system) and place of residence [26]. Under the *Hukou* scheme, SHI and other social welfare benefits are closely tied to the household registration system [27]. Disparities exist in the coverage of different types of SHI. Urban health insurance schemes like UEI and URI offer more comprehensive coverage than NCMS [28]. NCMS involves higher co-payments and does not provide as much financial protection as urban health insurance schemes [29].

In China, the SHI coverage and copayment rates of healthcare expenses for those who receive healthcare services outside their hometown vary among different provinces [30]. Even within the same province, there may be inconsistency regarding the reimbursement eligibility and copayment rates for prescriptions, outpatient and inpatient services, [30]. Due to data limitations, we were unable to compare the reimbursement rates between different provinces or cities. However, one commonality in receiving healthcare services outside OMWs’ hometown across different provinces is that the copayment rate of outpatient and inpatient expenses for UEI and URI beneficiaries are lower than that for NCMS recipients [25]. Rural-to-urban migrant workers often register their SHI in their hometown rather than their current place of residence [27]. Most rural-to-urban OMWs with SHI registered in their hometowns are covered by the NCMS [31]. In contrast, OMWs cannot enroll in NCMS if their SHI is registered in their current place of residence; instead, they are more likely to be covered by UEI or URI [31]. As UEI or URI offers greater financial protection compared to NCMS [25], OMWs with SHI registered in their current place of residence may derive more benefits from existing health insurance programs than those who do not. Therefore, this study did not examine the impact of SHI registered in different provinces on healthcare service utilization; instead, we focused on assessing the association between having SHI registered in their current place of residence and healthcare service utilization among OMWs.

Additionally, the Chinese government has initiated the direct settlement of inpatient expenses across provinces since 2016 [32]. In early 2021, the Chinese government launched the direct settlement for outpatient expenses across provinces in 12 pilot cities and expanded its coverage to other provinces in the following year [32]. However, although these interprovincial direct settlements streamline the reimbursement process, the copayment rates for healthcare expenses still depend on the patient’s type of SHI [30]. Therefore, the implementation of direct settlement for interprovincial healthcare expenses does not alter the fact that the copayment rate for UEI and URI remains lower than that for NCMS [30]. In this context, even though OMWs are eligible to use SHI registered in their hometown when seeking healthcare services at current place of residence, those with SHI registered in current place of residence may still benefit more in accessing healthcare services than those with SHI registered in their hometown.

Researchers have found that older migrants were less likely to use healthcare than natives, although this study did not specifically investigate OMWs [33]. Although researchers have identified a positive association between health insurance coverage and increased healthcare utilization among OMWs [34], the relationship between the registration location of SHI and healthcare use among OWMs has not been investigated. Previous research has emphasized that SHI schemes with geographical restrictions contribute to disparities in health outcomes [35]. However, there is limited research on the relationship between migration range and healthcare utilization among OMWs. The practice of limiting migration through administrative registration systems is not unique to China [36]. The administrative registration system in China may share similarities with migrant administration systems in many countries, where such systems regulate the extent of migration [37]. This study could provide empirical evidence that may benefit countries in the global community facing similar challenges.

## Methods

### Data Source

The data for this study were obtained from the China Migrant Dynamic Monitoring Survey (CMDMS), a national cross-sectional dataset. The CMDMS focused on migrating families and collected information about health insurance, healthcare utilization, as well as demographic and socioeconomic characteristics among Chinese OMWs [34]. The last wave of the CMDMS that collected information on healthcare utilization among OMWs was conducted in 2015 [31]. Although researchers have used the 2017 wave of CMDMS to investigate older migrants, they have not focused on OMWs [38]. Therefore, the sample for this paper includes OMWs who were interviewed in 2015. The survey employed a multi-stage, stratified, probabilistic sampling method, covering 32 provincial-level units [31]. The sampling process initially selected counties and then towns/streets, communities, and individuals, based on the statistics of migrants in the 2014 annual report of the China National Health Commission and the latest statistics of each provincial health commission [31]. This survey is considered one of the most representative national datasets for studying OMWs in China [31, 39]. The analysis focused on OMWs aged 60 years and above who migrated within mainland China (N = 3,050) and OMWs diagnosed with diseases or injuries requiring inpatient services (N = 175).

### Variable Specification

#### Dependent Variable

The two primary outcomes of interest are doctor visits for illness and the use of inpatient services. Doctor visits for illness are a dichotomized variable constructed from the question, “Do you visit doctors to seek timely healthcare treatment when you are sick?” Responses are coded as 1 for “yes” (indicating doctor visits) and 0 for “no” (no healthcare treatment or self-treatment without doctor visits) [40]. The use of inpatient use is a dichotomized variable based on the question posed to OMWs diagnosed with diseases or injuries requiring inpatient services: “Did you receive inpatient use in the previous year?” [41].

#### Independent Variable

The independent variables of interest include the registration place of SHI and migration range. The registration place of SHI is a categorical variable with three levels: hometown (the reference group), current place of residence, and other areas. Migration range is a categorical variable indicating the distance an OMW is from their hometown: migrated within the same city (the reference category), migrated to a different city, and migrated to a different province [41].

#### Covariate

Based on existing literature and data availability [41, 42], this study controlled the following variables: age, gender, highest educational level, equivalized household expenditure, marital status, migration duration, type of SHI, self-assessed health, and hypertension/diabetes. Details can be found in Supplementary Material.

### Statistical Analysis

The analytical approach employed in this study consists of three steps.

Step 1: This study applied individual weighting to the summary statistics concerning healthcare utilization across different health insurance and Hukou status categories among OMWs. Subsequently, we utilized chi-square tests to assess potential disparities in healthcare utilization, with respect to the registration place of SHI and migration range.

Step 2: To address the first research question, we constructed binary logistic regression models to investigate the relationships among SHI, migration range, and the likelihood of visiting doctors for illness or utilizing inpatient services. The model was specified as follows:

$$y=\text{logit }\left(p\right)=\beta_{0}+\beta_{1}\times P+\beta_{2}\times R+\sum_{m=9}^{m}\left(\beta_{m}\times C_{m}\right)$$ (1)

In Eq. 1, y represents the probability model of the outcome variable (visiting doctors for illness or inpatient services use) under specific exposure of the independent factors. P denotes the registration place of SHI, and R denotes the migration range. C_m_ denotes the control variables. β_0_ is the constant term. β_m_ are the coefficients of the respective regressors to be estimated from the model, signifying the natural logarithm of the odds ratio (OR) for the occurrence of an individual’s doctor visits for illness or inpatient services use in the presence of the independent variable compared to its absence. The ORs were computed along with 95% confidence intervals (CI).

Step 3: Furthermore, we reported the marginal effects of having SHI registered in the current place of residence and different migration ranges on visiting doctors for illness and inpatient services use. Marginal effects present the probability of doctor visits or inpatient services use across participants with SHI registered in the current place of residence or different migration ranges while holding all other variables constant [43].

Lastly, this study carried out robustness checks. We further included Hukou status as an additional control variable in the logistic models. This was done to mitigate the potential influence of Hukou status on the association between SHI registration place, migration range, and healthcare use [44]. *Hukou* status is a binary variable with two categories: agricultural *Hukou* (the reference group) and non-agricultural *Hukou*. All analyses were conducted using Stata 18.0.

## Results

Table 1 provides sample characteristics of the full sample of OMWs, OMWs who sought healthcare through doctor visits when ill, and OMWs in need of inpatient services. Individual weights were applied for the statistical summary. The average age of OMWs was 64 years, with 72% being male, 92% married, and 89% having secondary education or lower. Additionally, 54% of OMWs had moved to a different province. Among OMWs, 58% did not seek healthcare services through doctor visits when ill, and 24% of those with illnesses or injuries necessitating inpatient services did not utilize them. Both OMWs who sought healthcare services through doctor visits and those utilizing inpatient services for diagnosed illnesses/injuries) exhibited a higher proportion of individuals registered in their current place of residence, being enlisted in UEI, and holding non-agricultural Hukou status. Specifically, in comparison with the full sample, OMWs who sought healthcare services through doctor visits when ill seemed to have a higher proportion of individuals migrating to a different province. OMWs who used inpatient services for diagnosed illnesses/injuries seemed to have a lower proportion of individuals migrating to a different province and being covered by SHI.

**TABLE 1:** Weighted description of the sample (Migrants Dynamic Monitoring Survey, China. 2015).

Variables	Full sample	Visiting doctors when sick	OMWs in need of inpatient services
	Mean (SD)/Percentages		
Visiting doctor when sick			
no	57.78%		
yes	42.22%		
Inpatient services use			
no			24.40%
yes			75.60%
Registration place of SHI			
current place of residence	9.99%	11.79%	13.23%
hometown	89.75%	88.12%	85.71%
other areas	0.26%	0.09%	1.06%
Migration range			
migrated within the same city	16.76%	16.18%	26.10%
migrated to a different city	28.84%	26.89%	20.43%
migrated to a different province	54.40%	56.93%	53.48%
Age (years)	63.62 (3.47)	63.69 (3.43)	63.78 (3.37)
Male	72.11%	71.42%	77.10%
Highest educational level			
no formal education	14.25%	12.95%	8.34%
primary/secondary education	74.96%	72.24%	74.52%
high school or above	10.79%	14.82%	17.14%
Marital status			
unmarried	0.62%	0.23%	0.05%
married	92.00%	90.16%	93.87%
divorced/widowed	7.38%	9.61%	6.08%
Type of SHI			
no insurance	12.23%	11.85%	21.22%
NCMS	69.82%	66.68%	61.97%
URHI	4.97%	6.21%	0.99%
URI	6.30%	6.45%	4.87%
UEI	6.39%	8.14%	10.96%
other	0.29%	0.68%	0%
Non-agricultural *Hukou*	17.28%	21.76%	22.87%
Migration duration (years)	7.74 (6.88)	7.10 (6.67)	8.77 (6.58)
Self-assessed health			
healthy	63.08%	65.34%	32.31%
generally healthy	32.37%	29.82%	33.81%
unhealthy but can self-care	4.26%	4.44%	28.62%
cannot self-care	0.29%	0.04%	5.26%
Having hypertension/diabetes	12.47%	14.52%	43.09%
Equivalized monthly after-tax household expenditure (CNY)	1,504.58 (1,081.24)	1,629.44 (1,258.45)	1,614.93 (871.01)
*N*	3,050	1,184	175

SHI, social health insurance; NCMS, New Rural Cooperative Medical Scheme; URHI, Urban and Rural Health Insurance; URI, Urban Resident Insurance; UEI, Urban Employee Insurance. Migrants Dynamic Monitoring Survey, China, 2015.

Table 2 provides the rates of doctor visits for illness and inpatient services use based on the place of SHI registration and migration range. The rate of inpatient services use consistently exceeded the rate of doctor visits among individuals with different SHI registration places and migration ranges. However, it appears that there were no significant differences in the rate of doctor visits for illness and the utilization of inpatient services among OMWs based on the registration place of SHI and migration range.

**TABLE 2:** Rates of visiting doctors when sick and inpatient services use among older migrant workers by the registration place of social health insurance and migration range (Migrants Dynamic Monitoring Survey, China. 2015).

Variables	Visiting doctors when sick (%)	χ^2^	OMWs in need of inpatient services (%)	χ^2^
	Percentages (%)			
Registration place of SHI				
current place of residence	42.28	1.97	77.78	0.82
hometown	38.46		71.43	
other areas	41.67		100.00	
Migration range				
migrated within the same city	35.76	5.87*	74.65	0.10
migrated to a different city	38.31		72.50	
migrated to a different province	41.41		72.34	

SHI, social health insurance. *p < 0.05. Migrants Dynamic Monitoring Survey, China, 2015.

Table 3 presents the results of logistic regression models that investigated the relationship between SHI, migration range, and healthcare use (Supplementary Table S1 provides the full results). Regarding doctor visits, having SHI registered in the current place of residence was associated with a 47% increase in the odds of doctor visits when ill, compared to having SHI registered in their hometown (OR = 1.47, 95%CI = 1.12–1.93). Additionally, in the full sample, OMWs who migrated to a different province also had a higher likelihood of visiting doctors when ill (OR = 1.25, 95%CI = 1.00–1.55) compared to OMWs who migrated within a city. However, it appears that neither the registration place of SHI nor the migration range showed significant associations with the odds of inpatient services use.

**TABLE 3:** Binary logistic regression examining the association between social health insurance registration place, migration range, and healthcare use (Migrants Dynamic Monitoring Survey, China. 2015).

Variables	Visiting doctors	Inpatient services use
	Full sample	OMWs in need of inpatient services
	OR ^P^ (95%CI)	
Registration place of SHI (Ref: hometown)		
current place of residence	1.47**	1.27
	(1.12–1.93)	(0.36–4.45)
other areas	1.60	-
	(0.47–5.49)	
Migration range (Ref: migrated within the same city)		
migrated to a different city	1.11	1.12
	(0.88–1.39)	(0.34–3.71)
migrated to a different province	1.25*	1.06
	(1.00–1.55)	(0.40–2.83)
Controls	Yes	Yes
*N*	3,050	175

OMWs, older migrant workers; S.E., standard errors; SHI, social health insurance. *p < 0.05, **p < 0.01, ***p < 0.001. Migrants Dynamic Monitoring Survey, China, 2015.

Based on the findings from logistic regression models (Table 3), Table 4 presents the results of the average marginal effects, indicating the association among having SHI registered in the current place of residence, migration range, and healthcare use. In the full sample, on average, the likelihood of seeking healthcare treatment through doctor visits increased by 9% when SHI registration shifted from hometown to the current place of residence. Similarly, it increased by 5% when the migration range transitioned from within a province to moving to a different province. However, it seems that neither the registration place of SHI nor the migration range showed significant marginal effects on inpatient services use.

**TABLE 4:** Average marginal effects of social health insurance registration in the current place of residence and migration to a different province on healthcare utilization (Migrants Dynamic Monitoring Survey, China. 2015).

	Visiting doctors	Inpatient services use
	Full sample	OMWs in need of inpatient services
	Margin ^P^ (S.E.)	
Having SHI registered in current place of residence	0.09** (0.03)	0.04 (0.11)
Migrated to a different city	0.02 (0.03)	0.02 (0.11)
Migrated to a different province	0.05* (0.03)	0.01 (0.09)
Controls	Yes	Yes

OMWs, older migrant workers; S.E., standard errors; SHI, social health insurance. *p < 0.05, **p < 0.01, ***p < 0.001. Migrants Dynamic Monitoring Survey, China, 2015.

In the robustness check, this study further controlled for the *Hukou* status in the logistical models. The findings, as presented in Supplementary Tables S2, S3, were consistent with the main models: having SHI registered in the current place of residence and migration to a different province were associated with higher odds of seeking healthcare services through doctor visits when unwell.

## Discussion

This study investigated the influence of SHI registration place and migration range on healthcare utilization among OMWs. The findings indicate that having SHI registered in the current place of residence and migrating to a different province are significantly associated with a higher probability of OMWs seeking healthcare services through doctor visits.

The findings of this study suggest that having SHI registered in their current place of residence facilitated doctor visits when OMWs were sick. This may be attributed to several factors. Firstly, rural-to-urban OMWs with SHI registered in their current place of residence are more likely to enroll in urban health insurance programs, such as UEI, which offers higher financial protection than URI or NCMS. By contrast, OMWs with SHI registered in their hometown are more likely to be covered by URI or NCMS [40]. Hou and Zhang [45] found that rural-to-urban migrants who received urban health insurance had a higher likelihood to use healthcare than NCMS enrollees. Secondly, when seeking healthcare across different regions, the co-payment and maximum payment limits are determined based on the location where individuals have registered their SHI, rather than the location where they received healthcare treatment [30]. This can result in some services not being covered when seeking healthcare outside the registered SHI location, leading to a preference for local registration SHI [46]. Thirdly, the administration of SHI is under the governance of local authorities, making it challenging for OMWs to transfer SHI across regions [47]. Consequently, healthcare cost reimbursement for OMWs with SHI registered in their hometown may be less comprehensive than for those with SHI registered in their current place of residence.

Similarly, OMWs who migrated to a different province had a higher probability of seeking healthcare services compared to those who moved within the same province. This phenomenon can be attributed to several factors. In contrast to provincial migration, interprovincial migration often involves traversing longer geographical distances, a reduced likelihood of returning to the place of origin, less frequent commuting, and potentially prolonged periods of residence in the destination area. This protracted duration of residence in their current place of residence is more likely to lead to enrollment in the UEI. Empirical data in this study indicates a significantly greater prevalence of UEI coverage among OMWs who engaged in interprovincial migration compared to those who migrated within the same province. This aligns with previous research indicating that as the migration duration extends, rural-to-urban migrants are more likely to enroll in health insurance schemes at their current place of residence [48]. For international immigrants, previous studies also suggest the effect of health insurance on cross-border healthcare seeking is significantly influenced by migration distance [49]. Additionally, metropolitan areas tend to concentrate high-quality healthcare services and facilities. Consequently, interprovincial migration to major urban centers can enhance OMWs’ access to better healthcare services in their current place of residence, a particularly salient benefit for older OMWs who may have increased health needs due to their older age.

In contrast, it appears that neither SHI registration place nor migration range significantly influences the receipt of inpatient services among OMWs. This may be attributed to differences in demand elasticity between inpatient services and outpatient services [50]. Inpatient services demand is less responsive to price changes compared to outpatient services demand [51].

### Implications

Above all, the situation in China holds significant implications for the development of SHI in global communities. Evidence from China provides insights into designing a healthcare financing system in LMICs countries that are in the initial stage of SHI development. Governments in LMICs should consider implementing targeted policies within their health insurance programs to provide adequate protection for OMWs. Furthermore, this study recommends that the Chinese government further expand the coverage of direct reimbursement for cross-province healthcare services. Expanding its coverage could enhance access to healthcare services for a broader range of health conditions [52]. Additionally, we advocate for the adoption of a unified administration of SHI at the provincial level across the nation. Several provinces in China have successfully implemented such a unified governance model, ensuring consistent coverage and co-payment policies across different regions within the province [30]. However, some provinces still lack this unified approach [48]. Establishing nationwide SHI administration standards could contribute to greater equity in healthcare access for all residents, including OMWs.

### Limitations

Several limitations should be acknowledged. Firstly, the study utilized data from the 2015 CMDMS due to its status as the most recent nationally representative survey of Chinese OMWs. To the best of our knowledge, there is no alternative for nationally representative surveys for OMWs in China after 2015. Secondly, due to data availability, we cannot assess the reimbursement rates for healthcare expenses in provinces or cities. Instead, our focus was on investigating the association between having SHI registered in their current place of residence and OMWs’ healthcare service use. Thirdly, the study relied on cross-sectional data, limiting the ability to establish causality. Fourthly, future research with a larger sample of OMWs who require inpatient services would provide valuable insights.

### Conclusion

In conclusion, this study suggests that Chinese OMWs may derive fewer benefits from SHI in seeking healthcare treatment through doctor visits when their SHI is not registered in their current place of residence, compared to those with SHI registered in their current place of residence. However, the inpatient services use did not appear to be associated with the SHI registration place and migration range. These findings provide insights on other nations that also have issues with increasing migrant workers. This study calls for governments in LMICs to consider targeted policies within their health financing schemes to support the vulnerable OMWs. This study also suggests the Chinese government expand the coverage of direct reimbursement for cross-province outpatient services and consider unifying the SHI administration standards to reduce healthcare access disparities.
