# Supplementary material for: Slipping Through the Gap: Exploring the Influence of Social Health Insurance on Access to Healthcare for Older Migrant Workers
Source: Int J Public Health. 2024 Mar 13;69:1606655. doi: 10.3389/ijph.2024.1606655 (PMC10967954; doi:10.3389/ijph.2024.1606655)
Supplement: Supplementary file 1 [file DataSheet1.docx]

**Supplementary materials**

**Details of covariates**

Age is a continuous variable measured in years. Gender is a binary variable, with "female" as the reference category. The highest educational level is categorized into three groups: no formal education (the reference group), primary or secondary education, and high school education or above. Equivalized household expenditure is a continuous variable constructed based on the question ‘What is your monthly after-tax household expenditure’. This study adjusted this variable for household size using the equivalence scale method (1). Subsequently, we logarithmically transformed and winsorized it at the 0.5% quantile on both ends to mitigate the influence of extreme values (1). Marital status has three categories: unmarried, married (used as the reference group), and divorced/widowed. The type of SHI is a categorical variable with several categories: no SHI (the reference group), New Rural Cooperative Medical Scheme (NCMS), Urban and Rural Health Insurance (URHI), Urban Resident Insurance (URI), and Urban Employee Insurance (UEI). Migration duration is a continuous variable measured in years, also winsorized at the 0.5% quantile on both ends (1). Self-assessed health has four categories: healthy (the reference group), generally healthy, unhealthy but capable of self-care, and unable to self-care. Hypertension/diabetes is a dichotomized variable with "no" as the reference category, determined by the question, "Have you been diagnosed with hypertension or diabetes prescribed by doctors?".

**Table S1. Binary logistic regression examining the association between the registration place of social health insurance, migration range, and healthcare use (China. 2015).**

|  | **Visiting doctors** | **Inpatient services use** |
| --- | --- | --- |
|  | **Full sample** | **OMWs in need of inpatient services** |
| **Variables** | **OR ^P^(95%CI)** |  |
| **Registration place of SHI**  (Ref: hometown) |  |  |
| current place of residence | 1.47** | 1.27 |
|  | (1.12 - 1.93) | (0.36 - 4.45) |
| other areas | 1.60 | - |
|  | (0.47 - 5.49) |  |
| **Migration range** (Ref: migrated within the same city) |  |  |
| migrated to a different city | 1.11 | 1.12 |
|  | (0.88 - 1.39) | (0.34 - 3.71) |
| migrated to a different province | 1.25* | 1.06 |
|  | (1.00 - 1.55) | (0.40 - 2.83) |
| **Age** (years) | 1.02 | 1.00 |
|  | (0.99 - 1.05) | (0.90 - 1.11) |
| **Female** (Ref: female) |  |  |
| Male | 1.21 | 0.99 |
|  | (1.00 - 1.48) | (0.36 - 2.67) |
| **Education** (Ref: no formal education) |  |  |
| primary or secondary education | 1.02 | 0.41 |
|  | (0.78 - 1.34) | (0.11 - 1.56) |
| high school education or above | 1.64* | 1.15 |
|  | (1.10 - 2.44) | (0.11 - 12.41) |
| **Equivalized household expenditure** (Log) | 1.10 | 0.63 |
|  | (0.94 - 1.29) | (0.28 - 1.38) |
| **Marital status** (Ref: married) |  |  |
| Unmarried | 0.89 | - |
|  | (0.33 - 2.39) |  |
| divorced/widowed | 0.98 | 0.86 |
|  | (0.70 - 1.37) | (0.14 - 5.36) |
| **Migration duration** (years) | 0.98** | 1.07 |
|  | (0.97 - 0.99) | (1.00 - 1.15) |
| **Type of SHI** (Ref: no SHI) |  |  |
| NCMS | 0.78 | 0.98 |
|  | (0.54 - 1.11) | (0.09 - 11.00) |
| URHI | 0.74 | 0.31 |
|  | (0.53 - 1.04) | (0.07 - 1.39) |
| UEI | 0.94 | 2.62 |
|  | (0.67 - 1.33) | (0.40 - 17.22) |
| **Self-rated health** (Ref: healthy) |  |  |
| generally healthy | 0.75** | 0.60 |
|  | (0.63 - 0.91) | (0.20 - 1.76) |
| unhealthy but capable of self-care | 0.74 | 0.60 |
|  | (0.48 - 1.14) | (0.15 - 2.32) |
| unable to self-care | 1.36 | 0.95 |
|  | (0.21 - 8.61) | (0.07 - 12.43) |
| **Having hypertension/diabetes** (Ref: no) | 1.16 | 2.17 |
| Yes | (0.90 - 1.51) | (0.78 - 6.03) |
| *N* | 3050 | 175 |

OMWs, older migrant workers. S.E., standard errors. SHI, social health insurance. NCMS, New Rural Cooperative Medical Scheme. URHI, Urban and Rural Health Insurance. URI, Urban Resident Insurance. UEI, Urban Employee Insurance. **p* < 0.05, ***p* < 0.01, ****p* < 0.001. Migrants Dynamic Monitoring Survey, China, 2015.

**Table S2. Robustness checks: Binary logistic regression examining the association Between social health insurance registration, migration range, and healthcare use (China. 2015).**

|  | **Visiting doctors** | **Inpatient services use** |
| --- | --- | --- |
|  | **Full sample** | **OMWs in need of inpatient services** |
| **Variables** | **OR ^P^(95%CI)** |  |
| **Registration place of SHI**  (Ref: hometown) |  |  |
| current place of residence | 1.57** | 1.19 |
|  | (1.19 - 2.06) | (0.35 - 4.05) |
| other areas | 1.59 | - |
|  | (0.49 - 5.10) |  |
| **Migration range** (Ref: migrated within the same city) |  |  |
| migrated to a different city | 1.10 | 1.15 |
|  | (0.88 - 1.38) | (0.35 - 3.78) |
| migrated to a different province | 1.25* | 1.11 |
|  | (1.01 - 1.56) | (0.41 - 3.03) |
| Controls | Yes | Yes |
| *N* | 3050 | 129 |

OMWs, older migrant workers. S.E., standard errors. SHI, social health insurance. **p* < 0.05, ***p* < 0.01, ****p* < 0.001. Migrants Dynamic Monitoring Survey, China, 2015.

**Table S3. Robustness checks: Average marginal effects of social health insurance registered in the current place of residence and migration to a different province on healthcare utilization (China. 2015).**

|  | **Visiting doctors** | **Inpatient services use** |
| --- | --- | --- |
|  | **Full sample** | **OMWs in need of inpatient services** |
|  | **Margin ^P^ (S.E.)** |  |
| **Having SHI registered in current place of residence** | 0.11**(0.03) | 0.03(0.11) |
| **Migrated to a different city** | 0.02(0.03) | 0.02(0.11) |
| **Migrated to a different province** | 0.05*(0.03) | 0.02(0.09） |
| Controls | Yes | Yes |

OMWs, older migrant workers. S.E., standard errors. SHI, social health insurance. **p* < 0.05, ***p* < 0.01, ****p* < 0.001. Migrants Dynamic Monitoring Survey, China, 2015.

**References**

1. Long C, Chen F, Ye Y, Ji L, Xu X, Tang S. Inequalities in the access to health services among older migrants: evidence from the China Migrant Dynamic Monitoring Survey. International Journal of Public Health. 2023;68.
